# Lipid Droplets and *Mycobacterium leprae* Infection

**DOI:** 10.1155/2012/361374

**Published:** 2012-11-12

**Authors:** Ayssar A. Elamin, Matthias Stehr, Mahavir Singh

**Affiliations:** ^1^Department of Research and Development, LIONEX Diagnostics and Therapeutics GmbH, 38126 Braunschweig, Germany; ^2^Department of Genome Analytics, Helmholtz Centre for Infection Research, Inhoffenstrasse 7, 38124 Braunschweig, Germany

## Abstract

Leprosy is a chronic infectious disease and is a major source of morbidity in developing countries. Leprosy is caused by the obligate intracellular bacterium *Mycobacterium leprae*, which infects as primary target Schwann cells. Lepromatous leprosy exhibits multiple lesions of the skin, eyes, nerves, and lymph nodes. The sites of infection are characterized by the presence of foamy macrophages, fully packed with lipid droplets (LDs), which are induced by *M. leprae*. In the last years, it has become evident that *M. tuberculosis* imports lipids from foamy macrophages and is dependent on fatty acids for growth in infected macrophages. *M. leprae* seems to have similar mechanisms for scavenging lipids from the host. But due to the inability to culture *M. leprae* on laboratory media, research progresses only slowly. However, in the last years, substantial progress has been made in the field of lipid metabolism in *M. leprae*. Herein, we will present and summarize the lipid droplets formation and the metabolism of lipids during *M. leprae* infection.

## 1. Introduction

Leprosy, a major source of morbidity in developing countries, is a chronic infectious disease caused by the obligate intracellular bacterium *Mycobacterium leprae *[1, 2]. According to the system of classification of Ridley and Jopling (1966), leprosy patients show two major manifestations of the disease, designated as lepromatous leprosy (LL) and tuberculoid leprosy (TT) [1]. TT is observed in patients with good T-cell mediated (Th1) immunity and is characterized by granuloma formation and death of Schwann cells (SCs) leading to loss of myelin sheath and nerve destruction [3, 4]. TT shows onlyfewlesions, and bacillicanrarelybeidentified. Patients with poor T-cell mediated immunity show the lepromatous-type leprosy (LL). LL leads to massive bacterial load inside host cells specially SCs and macrophages [3, 5–7]. The lesions of TT and LL types are named as T-lep and L-lep lesions, respectively, but damage of the nerves is observed in most of the cases of both types [7]. 

Lepromatous leprosy exhibits multiple lesions of the skin, eyes, nerves, and lymph nodes, which are characterized by tumor-like accumulations of foamy macrophages. The foamy macrophages are fully packed with lipid droplets (LDs) and contain high numbers of lepra bacilli. These aggregations of foamy macrophages expand slowly and disfigure the body of the host [8]. Foamy macrophages were described first by the pathologist Rudolph Virchow [9] and despite these lipid-laden cells are a hallmark of lepromatous leprosy, only recently a few researchers started to elucidate the molecular biology of these cells. In the last ten years, it has become evident that *M. tuberculosis* induces the formation of foamy macrophages, a process which appears to be a key event in both sustaining persistent bacteria and release of infectious bacilli [10]. Moreover host lipids from LDs are regarded as substantial nutrient source for mycobacteria during infection. Middlebrook already demonstrated in the late 1940s that mycobacterial growth *in vitro* was enhanced by supplementation with oleic acid [11]. Wheeler et al. reported insufficient fatty acid synthase activity to support growth in *M. leprae*. This finding supported the hypothesis that *M. leprae* scavenges lipids from the host cell [12]. Over the last years, it has become evident that survival and persistence of *M. tuberculosis* is critically dependent on lipid body formation. Furthermore, lipid body formation seems to be the prerequisite for transition of *M. tuberculosis* to the dormant state. This goes along with the important observation that sputum from tuberculosis patients contains lipid body-laden bacilli [13, 14]. 

For a great number of mycobacterial species, intracellular lipid bodies were reported [15–25]. In the dormant state, lipids from lipid bodies appear to be the primary carbon source for *M. tuberculosis in vivo*. For *M. tuberculosis,* several bacterial genes are upregulated during the dormant state and have been reported to be involved in lipid metabolism such as diacylglycerol acyltransferase (tgs1), lipase (lipY), and isocitrate lyase (icl) [26, 27].

*M. leprae* has a small genome (3.2 Mb). The obligate intracellular organism shows a moderate genome degradation and several genes are absent when compared with other mycobacterial species. Due to the gene loss, *M. leprae* is strongly dependent on thehost for basic metabolic functions [3, 28]. Macrophages infected with *M. leprae* contain oxidized host lipids and it has been observed that *M. leprae* upregulates 13 host lipidmetabolism genes in T-leplesions and 26 in L-leplesions. The oxidized lipids inhibit innate immune responses and thus seem to be an important virulence factor for the organism [29]. 

This paper highlights the importance of the LDs as one of the most unique determinant for *M. leprae* persistence and virulence. The formation of LDs in *M. leprae* infected cells has been compared to LDs formation in macrophages infected with *M. tuberculosis*. Thereafter, we will focus on the interplay between the LDs of the host and the pathogen. 

## 2. *M. leprae* Induces Lipid Droplets Formation in the Host Cell 

*M. leprae* infects preferentially macrophages and SCs [6]. A typical feature of lepromatous leprosy is the survival and replication of *M. leprae* stored within the LDs in the enlarged phagosome of histiocytes. Due to strong immune response against *M. leprae* and arrested bacilli growth in T-lep lesions, the occurrence of foamy cells of *M. leprae*-infected macrophages/SCs is a marker only in L-lep lesions [5, 9]. In L-lep lesions, the expression of lipoprotein and fatty acid metabolism, including lipases genes, was 4-fold greater than in T-lep lesions which supports the previous observation [29]. Lipid droplets are thought to be an important nutrient source for the bacillus. A major concern in leprosy is peripheral neuropathy. The damage to nerves of the peripheral nervous system is caused by the infection of SCs by *M. leprae*. In LL nerve biopsies, highly infected SCs also contain LDs and show a foamy appearance, such as Virchow cells found in dermal lesions [30]. 

The biology of these foamy cells has been characterized poorly until now. Neither the origin nor nature of the lipids has been elucidated yet. Only recently, *in vitro* studies by Mattos could show that *M. leprae* induces the formation of LDs in human SCs [5]. Moreover, the group found that LDs are promptly recruited to bacterial phagosomes. In SCs, LDs recruiting by bacterial phagosomes depends on cytoskeletal reorganization and PI3 K signaling but is independent of TLR2 bacterial sensing [5].

**Figure 1:**
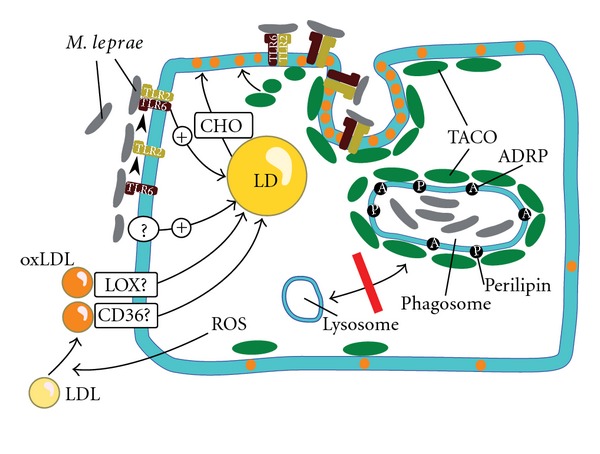
Basic mechanisms of lipid droplet biogenesis in macrophages infected with *M. leprae*. TLR mediated uptake of *M. leprae*: *M. leprae* attaches to TLR2 and TLR6. Heterodimerization of TLR2 and TLR6 induces downstream signalling and subsequent cholesterol accumulation by LDs formation [32, 33]. In SCs, TLR6, but not TLR2, is essential for *M. leprae*-induced LDs biogenesis [5]. Cholesterol accumulates at the site of mycobacterial entry and promotes mycobacterial uptake. Cholesterol also recruits TACO from the plasma membrane to the phagosome [34]. TACO prevents phagosome-lysosome fusion and promotes intracellular survival [35, 36]. Uptake by scavenger receptors (proven for *M. tuberculosis*, hypothetical for *M. leprae*): reactive oxygen species might oxidize low-density lipoprotein (LDL) to oxLDL, which is thought to be subsequently bound and taken up by scavenger receptors CD36 and LOX1. A: ADRP; CHO: cholesterol; P: perilipin. Unknown mechanisms for LDs induction are indicated with a question mark.

Important markers for the lipid accumulation in adipocytes or macrophages are lipid-droplet-associated proteins such as adipose differentiation-related protein ADRP and perilipin, which play essential roles in lipid-droplet formation [31]. After phagocytosis of live *M. leprae,* ADRP expression is constantly upregulated in human monocytes. ADRP and perilipin are localized at the phagosomal membrane (Figure 1) [31]. 

### 2.1. Receptor-Mediated LDs Formation

*Mycobacterium bovis* Bacillus Calmette-Guérin (BCG) and *M. leprae* are recognised by the Toll-like receptors (TLR) TLR6 and TLR2 [32, 37] and induces the formation of foamy macrophages [38]. For BCG it has been shown that surface molecule lipoarabinomannan (LAM) binds to TLR2. [39]. 

*M. leprae* association to macrophages is mediated by binding of the bacteria to TLR2 and TLR6. Heterodimerization of TLR2 and TLR6 leads to downstream signalling and subsequent LDs formation (Figure 1) [32, 33]. Macrophage association is not dependent on binding to TLR2 or TLR6. Neither a TLR2^−/−^ nor TLR6^−/−^ knockout macrophage shows reduced binding to *M. leprae*. This suggests that both TLR2 and TLR6 can bind *M. leprae* alone, or/and the presence of other receptors, binding to *M. leprae*. The TLR2^−/−^ or TLR6^−/−^ knockout macrophages do not also completely abolish LD formation, but show only reduced LDs formation [32]. This suggests the presence of additional signalling pathways for LDs formation. In contrast TLR6, but not TLR2, is essential for *M. leprae*-induced LDs biogenesis in SCs [5]. 

At least one additional factor is required for general phagocytosis, in mycobacterial infection. Members of the CD36 family have to be shown to be required for uptake of mycobacteria [40]. Macrophages infected with *M. tuberculosis* show an increased surface expression of the type 1 scavenger receptors CD36 and LOX1, which facilitate the uptake of oxidized host macromolecules including OxLDL (Figure 1) [41].

These findings are consistent with the observation that genes for ADRP and CD36 are upregulated in L-lep lesions, accumulated with LD enriched macrophages [29, 32, 42]. 

Macrophages generate and release reactive oxygen species (ROS) during activation of the respiratory burst upon infection with pathogenic bacteria. Oxidative stress results not only in damage to cellular structures but also to oxidation of fatty acids, such as low density lipoproteins (OxLDL) in granulomas. OxLDL binding to LOX-1 also increases reactive oxygen species (ROS) formation [41, 43]. The binding of OxLDL to type 1 scavenger receptors CD36 and LOX1 induces increased surface expression of both receptors, leading to uptake of OxLDL [41, 43, 44]. In addition, CD36 increases the uptake of *M. tuberculosis* by macrophages [40]. The increased rate of OxLDL uptake results in the accumulation of oxidized lipids, which finally leads to the formation of foamy macrophages (Figure 1) [41]. *M. tuberculosis* and *M. leprae* benefit from the accumulated OxLDL in the infected macrophage. OxLDL-laden lung macrophages show enhanced replication of intracellular *M. tuberculosis* compared to macrophages loaded with nonoxidized LDL [41]. The presence of oxidized phospholipids in *M. leprae* infected macrophages downregulates the innate immune response and contributes to pathogenesis [29]. Moreover, scavenger receptor-deficient phagocytes are characterized by a reduced intracellular bacterial survival and a lower cytokine response [45].

### 2.2. Lipid Composition in the Foamy Macrophage

In 1863, Virchow described lipid-laden macrophages. The lipids in these foamy cells form droplets and surround also *M. leprae* within the phagososomes [46, 47]. This lipid layer or capsule forms a characteristic electron-transparent zone. In contrast to *M. tuberculosis*, lipid inclusions (lipid bodies) seem to be rather exceptional in *M. leprae* [47]. The electron-transparent zone contains mycoserosoic acids of phthiocerol dimycocerosates as well as phenolic glycolipids [48, 49]. Brennan reported the full characterization of three phenol-phthiocerol triglycosides by *M. leprae* [46]. It has been postulated that many of these same molecules together with phosphatidylinositol mannosides and phospholipids are released from the cell wall after synthesis, forming the capsule-like region [6]. The dominant lipid in the cell wall which gives *M. leprae* immunological specificity is phenolic glycolipid-1 (PGL-1). Phenolic glycolipid 1 has been isolated in relatively high concentrations from purified bacteria and from *M. leprae* infected tissues [50]. PGL-1 is thought to be a major component of the capsule in *M. leprae* and constitutes an important interface between bacteria and host [51]. It has been suggested that PGL-1 is involved in the interaction of *M. leprae* with the laminin of SCs, thus PGL-1 might play a role in peripheral nerve-bacillus interactions [52]. Moreover, phenolic glycolipids seem to be involved in the stimulation of suppressor T-cells in lepromatous leprosy [53]. Daniel et al. demonstrated that *M. tuberculosis* inside foamy macrophages imports fatty acids derived from host TAG and incorporates them intact into bacterial TAG, which is accumulated in lipid bodies [54].

Recently, it was reported that also LDs from *M. leprae* infected SCs and macrophages accumulate mainly host-derived lipids, such as oxidized phospholipids [29]. BODIPY stains infected SCs, indicating that LDs contain neutral lipids, such as triacylglycerols (TAG) [5].

### 2.3. Regulation of Host-Signaling by *M. leprae* Induced-Lipid Droplets

In *M. leprae* infected-cells, LDs show high ability to fuse further to form giant LDs. In most mammalian cells, dynein and extracellular signal-regulated kinase-2 (ERK2) facilitate LDs move along the cytoskeletal network [55]. Extracellular signal-regulated kinase-1/2 is one of the most upregulated signals after *M. leprae* induced-SCs demyelination and nerve injury [56]. Using NIH 3T3 cells, Andersson et al. demonstrated that ERK2 is an essential signal in LDs formation [57] and also seem to be crucial for the phospholipase D1- (PLD1-) mediated increases of LDs. The ERK2 mechanism is mediated by phosphorylation of dynein, which finally upregulates its association in ADRP-containing LDs [57]. 

Lipid droplets induction in all type of cells associates with lipid droplets protein adipophilin (ADFP) [58]. The classic model of LDs postulates that LDs formation take place in the outer leaflet of the endoplasmic reticulum (ER) membrane (the place of TAG synthesis). In this formation, the function of AFDP has been proposed in fatty acid absorption and transport. Cruz et al. showed that ADFP is overexpressed in L-lep lesions [29]. Furthermore, the authors also pinpointed similarity in overall observations to one of the important metabolic disease atherosclerosis. Similar observation in atherosclerosis showed that ADFP is the most expressed LD-associated protein found in macrophages and that the accumulation of LDs in lesional macrophages is associated with ADFP aggregation [58]. 

Several reports have supported the inflammatory role of macrophages and SCs, where LDs induction by these cells associated with modulation in production of inflammatory mediators [59–63]. Moreover, increased LDs formation accompanied by PGE2 and transforming growth factor-*β* (TGF-*β*1) synthesis in BCG infected macrophage while strong PGE2 was seen in foamy macrophages during *M. tuberculosis* infection [64, 65]. Cytokines, chemokines, and growth factors seem to be correlated with LDs formation by *M. leprae*. For instance, Persson et al. found that IL-1*β* and TNF-*α* inflate foamy macrophages formation [66]. *M. leprae* infection significantly increased NF-*κ*B, TNF-*α*, and IL-1*β* expression in NOD1- and NOD2-transfected macrophages cells [67]. Another example, IL-10 and IL-12 secretion correlated with bacilli-LD induction in L-lep lesions [32, 68]. Schwann cells are able to produce several type of cytokines, including IL-1, IL-6, IL-8, IL-10, IL-12, PGE2, TGF-*β*, and TNF-*α* [37, 69, 70]. PGE2 and IL-10 overexpression is associated with clog of IL-12 and NO production in *M. leprae*-infected SCs [32, 68]. In late infection state, *M. leprae* impede the expression of TNF signaling and induce expression of 9-O-acetyl GD3 as smart strategy to avoid the cellular apoptosis and facilitate its persistence inside the favored cell niche [37, 67, 71, 72]. Obviously, lipid biosynthesis and cytokine production as the whole immune response to *M. leprae* infection are well connected in SCs [5]. Innate immune response in infected SCs seems to be dependent on LDs accumulation and TLR2/TLR6 signaling. Using a nonsteroidal anti-inflammatory drug and fatty acids synthesis inhibitors, both LDs formation and innate immune responses completely abrogated, supporting the role of bacilli-LDs induction and immune responses [32, 37]. Another modulation observed is downregulation of Th1 responses and bactericidal activity through overproduced PGE2 where TLR2 and TLR6 heterodimerization plays important role [32]. In general, cytokine profile in both lesions well linked with TLRs function. *M. leprae *modified-TLRs function stimulates monocytes and macrophages in TT and LL disease type, but in L-lep lesions the adaptive response was debilitated more than what was seen in T-lep lesions [6]. Despite the strong evidences that LDs induction is associated with modulation of cytokines, it is not known whether cytokines signaling functions within LDs themselves.

## 3. Lipid Metabolism in *M. leprae* and *M. tuberculosis*

Due to the genome reduction *M. leprae* is strongly dependent on catalytic activities provided by the host [3, 28]. Macrophages infected with *M. leprae* contain oxidized host lipids and it has been observed that *M. leprae* upregulates 13 host lipidmetabolism genes in T-leplesions and 26 in L-leplesions [29]. But still it is not clear what carbon source *M. leprae* uses under aerobic or anaerobic conditions. The genome contains genes for utilization of sugars, such as glycolysis, tricarboxylic acid cycle (TCA), and monophosphate shunt (HMP) pathways. The *M. leprae* genome contains all genes for a functional glyoxylate cycle, which would enable the organism to use acetyl-CoA from the *β*-oxidation of lipids. *M. tuberculosis* derives much of its energy from the degradation of host-derived lipids [73, 74]. Neutral lipids are hydrolyzed by lipases or esterases, yielding fatty acids for energy generation and anabolism of membrane phospholipids. The genome of *M. tuberculosis* H37Rv contains twenty genes designated as putative lipases (*lipA* to *W*, except *K* and *S*) [74, 75]. In the *M. leprae* genome, only 2 lipase genes (*lipG*, *lipU*) were found, suggesting a gene loss in comparison to *M. tuberculosis*. But recently it has been found that in *M. tuberculosis* genome only six Lip expressed-enzymes showed reasonable hydrolase activity for long-chain triacylglycerols (LipY, LipC, LipL, LipX, LipK, and LipG). LipY (*Rv3097c*) has the highest activity, compared to all Lip enzymes. LipG and LipU from *M. leprae* are homologous with LipG and LipU from *M. tuberculosis* and show sequence identities of 72% and 79%, respectively. The lipases LipG and LipU from *M. tuberculosis* show very low and no activity with long-chain triacylglycerols as substrates [76]. *M. tuberculosis* LipY is suspected to be a major functional lipase, which utilizes stored triacylglycerols (TAG) during dormancy and reactivation of the pathogen [76, 77]. LipY shows only a weak similarity with *M. leprae* LipU (23% identity). In summary, it appears that *M. leprae* uses different lipases for the hydrolysis of fatty acids than *M. tuberculosis*. All *M. tuberculosis* and their *M. leprae* homologs enzymes involved in lipid metabolism in lipid bodies are summarized in Table 1. 

*M. tuberculosis* can grow on fatty acids as sole carbon source and it has been demonstrated that fatty acid oxidation is important for survival of the pathogen in the lungs of mice [83, 84]. Fatty acids are oxidized via the *β*-oxidation cycle and the glyoxylate shunt, to replenish TCA cycle intermediated during growth [88]. The *β*-oxidation cycle consists of five biochemical reactions, where one molecule acetyl-CoA of the fatty acid is split off per cycle. The genome of *M. tuberculosis* encodes around 100 genes, designated as *fad* genes (fatty acid degradation) with putative roles in the *β*-oxidation of fatty acids. While *E. coli* has only one enzyme for each step of the *β*-oxidation cycle, *M. tuberculosis* seems to have several backup enzymes for each reaction [89]. *M. leprae* has approximately one-third as many potential fad enzymes. However, there are three times more FadD acyl-CoA synthases than there are FadE acyl-CoA dehydrogenases, whereas these are predicted in equal amounts in *M. tuberculosis* [74]. The initial step of *β*-oxidation is the formation of acyl-CoA from free fatty acids and coenzyme A and is catalyzed by acyl-CoA synthase. The Acyl-CoA synthase *Rv1683* is suspected to be essential for TAG hydrolysis and growth [80, 90]. 

Even though *M. leprae* genome contains less necessary *β*-oxidation cycle genes than *M. tuberculosis*, transcripts analysis revealed active expression of acyl-CoA metabolic enzymes including *echA1* (ML0120, putative enoyl-CoA hydratase), *echA12 *(ML1241, possible enoyl-CoA hydratase), *fadA2* (ML2564, acetyl-CoA-acetyltransferase), *fadB2* (ML2461, 3-hydroxyacyl-CoA dehydrogenase), *fadD19* (ML0352, acyl-CoA synthase), *fadD26* (ML2358, fatty acid-CoA-ligase), *fadD29* (ML0132, probable fatty-acid-CoA synthetase), *fadD28* (ML0138, possible fatty-acid-CoA synthase), *fadE25* (ML0737, probable acyl-CoA dehydrogenase), and *fadE5* (ML2563, acyl-CoA dehydrogenase) [91, 92]. Combing observations from leprosy lesions, this gives strong evidence that host lipids provide the main carbon and energy sources for *M. leprae *during infection.

Together with malate synthase, isocitrate lyase (ICL) is the key enzyme of the glyoxylate cycle that catalyzes the cleavage of isocitrate to glyoxylate and succinate [88, 93]. The *M. leprae* genome contains a gene, coding for an isocitrate lyase, *aceA*. *AceA* is upregulated in both *M. leprae*-infected nude mouse and human lesions. [91]. The amino acid sequence of *aceA* (ML1985c) shows 80% identity with its homologue from *M. tuberculosis* ICL2 (*Rv1915*/*1916*). A second *icl* gene is not present in *M. leprae*, as observed in *M. tuberculosis*. This finding is of particular interest, because two *M. tuberculosis* isocitrate lyases, *icl *and* icl2*, are jointly required for in vivo growth and virulence [83, 84]. Deletion of *icl1* or *icl2* has little effect on bacterial growth in macrophages [83]. So far the evidence indicates that *M. leprae aceA* might play a slightly different role in both isocitrate lyases in *M. tuberculosis*.

## 4. *M. leprae* Has a Reduced Number of Triacylglycerol Synthase Genes 

**Figure 2:**
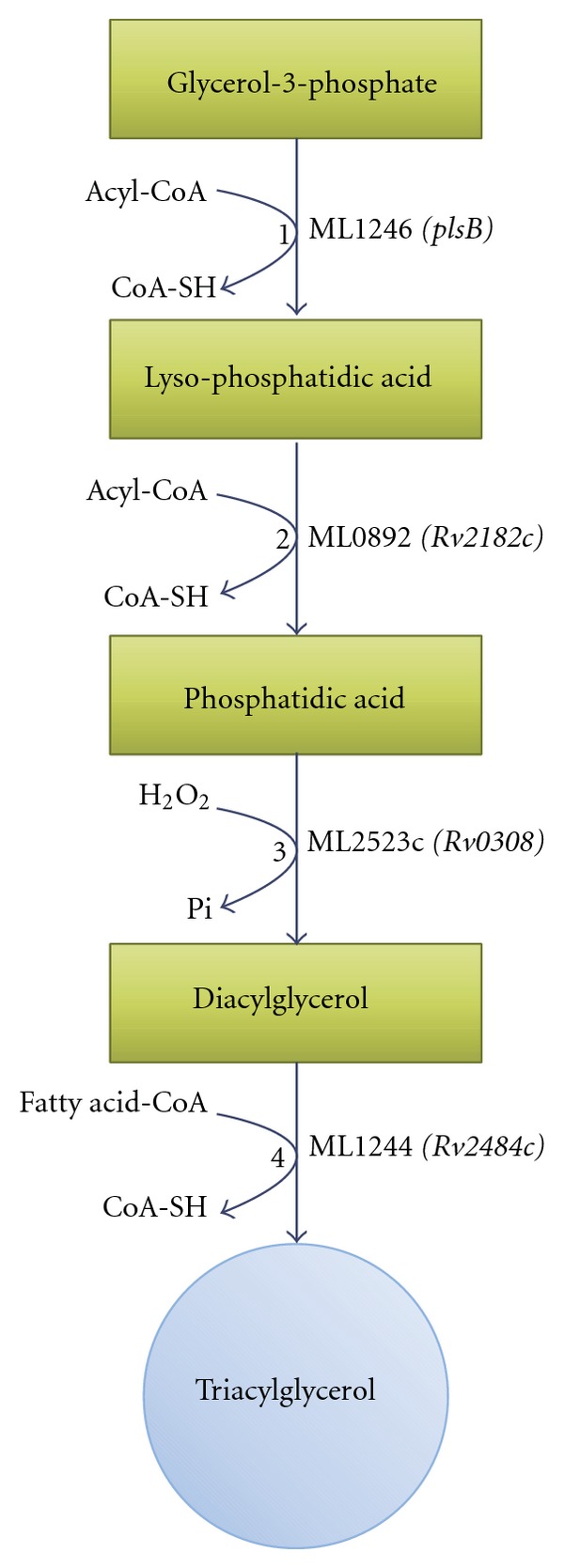
Schematic representation of putative genes involved in TAG biosynthesis in *M. leprae. *As a precursor, glycerol-3-phosphate further processed by sequential acylation steps, until last the transesterification of diacylglycerol (DAG) to triacylglycerol (TAG). Reaction 1 mediated by glycerol-3-phosphate acyltransferase, 1-acylglycerol-3-phosphate acyltransferase mediates reaction 2, phosphatidc acid phosphatase processes reaction 3, and the last reaction 4 is mediated by acyl-CoA: diacylglycerol acyltransferase enzyme.

For persistent *M. tuberculosis,* TAGs seem to be the major carbon and energy source. Biosynthesis of TAG consists of the sequential esterification of the glycerol moiety with fatty acyl-residues by various acyltransferases. The fatty acids are supplied by de novo biosynthesis or *β*-oxidation. Esterification occurs via sequential acylation of the sn-1,2 and 3 positions of glycerol-3-phosphate, and removal of the phosphate group before the last acylation step. The terminal reaction is the esterification of diacylglycerol (DAG) with acyl-CoA by an diacylglycerol acyltransferase (Figure 2) [94]. Bacteria do not contain DGATs but only bifunctional wax ester synthase/acyl-CoA: diacylglycerol acyltransferases (WS/DGAT). WS/DGATs mediate next to TAG formation the synthesis of waxes by esterification of acyl-CoA with alcohol [95]. The genome of *M. tuberculosis* codes for 15 genes which contain the highly conserved putative active site motif of WS/DGATs (HHxxxDG). These genes were designated as “*tgs*,” triacylglycerol synthases, but have only a weak sequence similarity to other WS/DGAT sequences. All 15 expressed mycobacterial tgs proteins show diacylglycerol acyltransferase activity and *tgs1* has the highest activity of all enzymes [26]. *Tgs1* appears to be a major contributor to TAG synthesis in *M. tuberculosis* so far [26, 78]. And moreover, two homologue proteins to *tgs1* and *tgs2* (BCG3153c and BCG3794c) and another poorly characterized acyltransferase (BCG1489c) were found to be exclusively associated to lipid bodies. Disruption of the *tgs* genes BCG3153c, BCG3794c, and BCG1489c reduces TAG accumulation during hypoxia-induced nonreplicating state, revealing that the enzymes are involved in TAG synthesis during latency and pathogenicity [80].

Ten of the 15 *tgs* genes in *M. tuberculosis* are located adjacent or proximal to 11 lip genes that are annotated as probable phospholipases or lipases-esterases carboxylesterases. Some *tgs* genes may be cotranscribed with neighboring lip genes and may synthesize triacylglycerols from the released fatty acids from the host [26]. Lip gene products may be important for utilization of TAGs during dormancy and upon reactivation after dormancy. The *tgs* gene *Rv0221* is located near LipC (*Rv0220*), lipW (*Rv0217c*), acyl-CoA synthetase (*Rv0214*), acyl-CoA dehydrogenase (*Rv0215c*), and an integral membrane acyltransferase (*Rv0228*). This clustering of genes of the fatty acid metabolism suggests that these genes may be cotranscribed and may release fatty acid from host TAG, carry out the transport of fatty acids and finally catalyze the resynthesis of TAGs in the pathogen. *Rv0221* and LipC have to be shown to be catalytical active [26, 76]. 

The *M. leprae* genome shows only one predicted gene product which has a significant degree of identity to any tgs enzymes from *M. tuberculosis *[26]. The* tgs* gene product ML1244 shows 72% identity to *Rv2484c* from *M. tuberculosis*. *Rv2484c* is located next to a carboxylesterase lipQ, (*Rv2485c*), a probable glycerol-3-phosphate acyltransferase, (*Rv2482c*), a lysophosphatidic acid acyltransferase-like protein (*Rv2483c*), and a probable enoyl-CoA hydratase (*Rv2486*). The gene cluster of lipid metabolism genes suggests a possible involvement of the gene products in the synthesis of TAG [26]. A few *tgs *genes (*Rv3234c*, *Rv3233c*, *Rv2285*, and *Rv1425*) are located proximally to lipoproteins, which may serve as donors or acceptors of fatty acids [26].

Ag85A, a mycoly transferase that is known to catalyze the formation of the cord factor was recently found to have additional DGAT activity [79]. The homologue gene of *M. tuberculosis Ag85A *is expressed in *M. leprae*. Although, mycoly transferase 85 complex genes (A, B, and C) transcripts were one of the major secreted group during mycobacterium infection of the mouse, *Ag85A* is one of the most genes shown to be relatively highly expressed either in infected nude mouse or human skin lesions [91].

## 5. Cholesterol Accumulation in the Host Cell and Utilization by *M. leprae*

Macrophages, infected with *M. leprae,* alter the lipid metabolism. Especially an increased accumulation of cholesterol and cholesterol esters has been found [42]. The increased cholesterol level was also associated with a lower level of esterase activity in infected macrophages [42].

Mattos et al. recently showed by high-performance thin-layer chromatography that the amount of cholesterol increases in SCs upon infection with *M. leprae *[5]. Cholesterol utilization was also identified to be required for mycobacterial persistence [96]. In 2008, Pandey and Sassetti found that *M. tuberculosis* can grow using cholesterol as a primary carbon source and that the *mce4* transporter is required for cholesterol uptake. 

While the *M. tuberculosis* genome contains four homologous mce operons, mce1-mce4, which are thought to encode lipid transporters [96, 97], *M. leprae* genome encodes only one operon (mce1). All *M. leprae* five *mce* genes were overexpressed during intracellular growth in mouse and human biopsies [91, 92]. This observation suggests that the intracellular bacilli population induces cholesterol uptake of the infected cell and subsequently uses the stored cholesterol as carbon and energy source. 

Cholesterol is also essential for survival of mycobacteria in macrophages. Cholesterol accumulates at the site of mycobacterial entry in macrophages and promotes mycobacterial uptake. For *M. tuberculosis* and *M. leprae* it has been shown that cholesterol mediates the recruitment of TACO from the plasma membrane to the phagosome [34]. TACO, also termed as coronin-1A (CORO1A), is a coat protein that prevents phagosome-lysosome fusion and thus degradation of mycobacteria in lysosomes [34, 35]. The entering of mycobacteria at cholesterol-rich domains of the plasma membrane and their subsequent uptake in TACO-coated phagosomes promotes intracellular survival [35, 36].

## 6. Conclusions

Studying *M. leprae* metabolisms during infection is as complex as the choice of invasion mechanisms traffic and as their multifarious factors for entry are. In this scenario, most of publications emphasize that leprosy maintains metabolic remodeling between both host and pathogen. A hallmark of intracellular infection is the formation of foamy host cells. Several results support the view that *M. leprae* actively catabolizes fatty acids for energy and produces a wide array of secretory proteins. Huge gaps remain in our knowledge of the structure and function of *M. leprae* lipid bodies, as the host metabolism required alterations to switch the organism to the utilization of lipids as alternate carbon sources. Investigating the metabolic aspects of *M. leprae* especially LDs formation is an important topic to learn more about metabolic pathways that could potentially be exploited for developing novel drugs against leprosy and tuberculosis.

## Figures and Tables

**Table 1:** Comparison between the enzymes involved in TAG metabolism in *M. tuberculosis* and *M. bovis* BCG and their homologous in *M. leprae*. ND, not determined.

Gene	Enzyme	Diacylglycerol acyltransferase activity (*in vitro*)	Influence on lipid bodies/TAG accumulation	*M. leprae* homologue	*M. leprae* homologue-diacylglycerol acyltransferase activity (*in vitro*)	Reference
*Rv3130c* (*tgs1*)	DGAT	+	Δ*tgs1*decreases TAG accumulation	ML1244	ND	[26, 78]
*Rv3734c* (*tgs2*)	DGAT	+	ND	ML1244	ND	[26, 78]
*Rv3234c *(*tgs3*)	DGAT	+	ND	ML1244	ND	[26, 78]
*Rv3088* (*tgs4*)	DGAT	+	ND	ML1244	ND	[26, 78]
*Rv1760 *	DGAT	+	ND	ML1244	ND	[26, 78]
*Rv2285 *	DGAT	+	ND	ML1244	ND	[26, 78]
*Rv3804c *(*85A*)	DGAT	+	Overexpression increases production of lipid bodies	ML0097 (85A)	ND	[79]
*BCG1489c* [*Rv1428c*]	DGAT	ND	Δ*BCG1489c* reduces TAG accumulation	ML2427c	ND	[80]
*BCG3153c* (*tgs1*) [*Rv3130c*]	DGAT	ND	Δ*BCG3153c* reduces TAG accumulation	ML1244	ND	[77]
*BCG3794c* (*tgs2*) [*Rv3734c*]	DGAT	ND	Δ*BCG3794c* reduces TAG accumulation	ML1244	ND	[80]

Gene	Enzyme	TAG-hydrolyzing activity (*in vitro*)	Influence on lipid bodies/TAG accumulation	*M. leprae* homologue	*M. leprae* homologue-TAG-hydrolyzing activity (*in vitro*)	Reference

*Rv3097c* (*lipY*)	Lipase/esterase	+	Δ*lipY* reduces TAG hydrolysis. Overexpression increases TAG hydrolysis	ML0314c (*lipU*) ML1053 ML1183c	ND	[76]
*Rv1399c* (*lipH* )	Lipase/esterase	+	ND	ML0314c (*lipU) *	ND	[75]
*BCG1721* [*Rv1683*]	Lipase/esterase	+	+	ML1346	ND	[80]
*Rv0183 *	Lipase/esterase	Hydrolyzes only monoacylglycerides	ND	ML2603	ND	[81]
*Rv2224c *	Lipase/esterase	ND	ND	ML1633c	ND	[82]

Gene	Enzyme	Isocitrate cleavage (*in vitro*)	Influence on lipid bodies/TAG accumulation	*M. leprae* homologue	*M. leprae* homologue-isocitrate cleavage (*in vitro*)	Reference

*Rv0467* (*icl*) *Rv1916* (*icl2*)	isocitrate lyase	+	ND (but the Δ*icl*, Δ*icl2 *strain shows no intracellular replication)	ML1985c (aceA)	ND	[83–87]
